# Supplementary material for: Using Co-Production to Develop “Sit Less at Work” Interventions in a Range of Organisations
Source: Int J Environ Res Public Health. 2021 Jul 22;18(15):7751. doi: 10.3390/ijerph18157751 (PMC8345370; doi:10.3390/ijerph18157751)
Supplement: Supplementary file 1 [file ijerph-18-07751-s001.zip › Devel Paper_Figure S1.pdf]

Development

Understand local barriers and facilitators to participation, e.g.:

- Nature of work, workload, time, individual working style, perceived impact on productivity
- Feeling self-conscious / distracting others
- Physical health, stress
- Peer support, social norms, office culture
- Existing work environment
- Upfront costs

Identification and use of theoretical model to operationalise intervention strategies, e.g.:

- Socio-Ecological Model, Social Cognitive Theory, the COM-B model (Capability, Opportunity, Motivation), Rogers' Diffusion on Innovations Theory, Theory of Planned Behaviour

Implementation

Embed intervention within local policies / strategies:

- Management consultation – gain high-level buy-in to support intervention launch / maintenance
- Understand and align with organisation's priorities / image – requires an in-depth understanding of the organisation processes and structures relevant to implementation
- Intervention needs to become institutionalised within the organisation to allow sustained adoption of intervention

Iterative and phased implementation:

- Use feedback from pilot study and participatory approach to further refine intervention and inform implementation

The use of a participatory / collaborative approach (consider establishing a workplace sitting committee):

- Employees – promote a sense of ownership via a bottom-up approach e.g. using workshops / focus groups
- Management – need support / engagement, formalise commitment, develop partnership between researchers and management e.g. involving organisation departments to support implementation logistics; management trialing intervention, distributing intervention emails, providing consent for staff to participate

Conduct a feasibility or pilot study within the target organisation:

- To support the understanding of the workplace facilities, routines, and the organisational structures, processes and culture and to further refine intervention development and implementation

Develop and implement an action plan incorporating the following key intervention characteristics:

- Flexible, can adapt to different groups of staff
- Provide a menu of strategies
- Target multiple levels of influence (individual, social, organisational, environmental)
- Low resource intensive
- Workplace champions to advocate and influence
- Consider interrupting sitting vs replacing prolonged sitting with standing/moving
- Tailor intervention to the needs of employees and organisation – ensuring it is applicable and relevant

Evaluation

Process – quantitative and qualitative measures:

- Mechanisms / process of change for organisations to understand e.g.:
  - Individual motivation, preference, interests, priorities
  - Personal challenge
  - Curiosity to try something new
  - Initial engagement then tailing-off
  - Task- and time-based routines, developing habits – certain tasks are easier to complete whilst not sitting; time can act as a trigger e.g. moving away from desk once per hour
  - Raise individual / organisational awareness
  - Encouragement from peers / management
  - Relevance to organisation's priorities
- Assessment of feasibility and acceptability
- Assessment of intervention fidelity
  - Delivered as planned
  - Assessment of harms / unintended consequences

Outcome – quantitative and qualitative measures:

- Health – physical (mainly musculoskeletal reported) and mental
- Sitting less or standing/moving more
- Work-related - productivity, concentration, energy levels
- Socio-environment changes
- Staff morale and autonomy / sense of empowerment
- Long-term cost-effectiveness analysis / return on investment
- Compensation effect i.e. when less sedentary at work, this is then compensated for by being more sedentary at home and vice versa

Take into account the real-world context to support knowledge translation, intervention generalisability and the potential for scaling-up the intervention:

- Have a good understanding of the local organisation processes and structures and wider policy issues
- Provide a detailed description of intervention development, implementation and evaluation processes to allow replication of methods

Figure S1. Operational framework for the development, implementation, and evaluation of interventions to reduce workplace sitting time (previously published [26])
