# Supplementary material for: Using Co-Production to Develop “Sit Less at Work” Interventions in a Range of Organisations
Source: Int J Environ Res Public Health. 2021 Jul 22;18(15):7751. doi: 10.3390/ijerph18157751 (PMC8345370; doi:10.3390/ijerph18157751)
Supplement: Supplementary file 1 [file ijerph-18-07751-s001.zip › Devel Paper_Table S1.pdf]

Table S1. Organisation characteristics (previously published [34])

Characteristic	Small business	Charity	Local authority	Large corporation
**Total number of employees**	8	488	4,146	119,300*
**Sector**	Private	Voluntary	Public	Private
**Description of organisation**	Provides information technology support to a range of other businesses and comprised one Managing Director, two managers and five employees.	Manages homes, and provides care and support to vulnerable people. Departments include: Directors, Computer Services, Finance, Customer Services, Human Resources, Performance and Central Services, New Business, Housing Services, and Property Services.	Set in a Metropolitan Borough. Work covers four Directorates: Adults, Health and Wellbeing; Corporate Resources; Learning and Opportunities; and Regeneration and Environment.	Banking business. Only one branch participated. This branch had both business and corporate teams and comprised 25 staff and two team leaders.
