# Supplementary material for: Using Co-Production to Develop “Sit Less at Work” Interventions in a Range of Organisations
Source: Int J Environ Res Public Health. 2021 Jul 22;18(15):7751. doi: 10.3390/ijerph18157751 (PMC8345370; doi:10.3390/ijerph18157751)
Supplement: Supplementary file 1 [file ijerph-18-07751-s001.zip › Devel Paper_Table S2.pdf]

Table S2. Intervention planning proforma

Action	Content	Timing	Management involvement	Barriers	How to overcome barriers	Who to action
