# Supplementary material for: Using Co-Production to Develop “Sit Less at Work” Interventions in a Range of Organisations
Source: Int J Environ Res Public Health. 2021 Jul 22;18(15):7751. doi: 10.3390/ijerph18157751 (PMC8345370; doi:10.3390/ijerph18157751)
Supplement: Supplementary file 1 [file ijerph-18-07751-s001.zip › Devel Paper_Table S3.pdf]

Table S3. Outputs from Workshop 1 for each organisation by level of influence

Ideas	Small business	Charity	Local authority	Large corporation
**Intrapersonal**	• Lunch trip by foot<br>• Walk to shop / Tesco<br>• APP-alarm walk<br>• Stand-up regularly<br>• Stand-up when on a phone call<br>• Make office manager coffee every 2 hours<br>• Break every hour<br>• Raise awareness<br>• Switch monitor off<br>• Lying down<br>• Run a mile	• Podcasts / voice memos vs docs / emails – walk and listen<br>• Personal behaviour / self-discipline – challenge yourself to move away from your desk for 10 minutes every hour<br>• Seek out people at their desk rather than email or phone – keep the door open your office to facilitate this<br>• Personal targets for steps/day and track activity using FitBit, or pedometers, record steps to see benefit of "walking / standing day"<br>• Walk to work 1 day a week or getting off public transport earlier and walking the rest<br>• Take regular short breaks<br>• Use the stairs and not the lift<br>• Make your own drinks<br>• Lunch outside office, walk to get lunch (don't bring your own food all the time)<br>• Get out of the office more	• Walk over to talk to people<br>• Don't always book meeting rooms on the floor that you work on<br>• Walk up and down a floor for exercise during the day<br>• Over lunch go for a walk<br>• Use stairs instead of lift<br>• Join exercise class / informal exercise gear in the atrium<br>• App on phone – if not moved, you get a prompt, reminder to get up regularly<br>• Stand on a regular basis<br>• Walk to meetings locally<br>• Active travel to work – walk / cycle – if you live too far away, get off a few stops early	• Use stairs not lift<br>• Take action with FitBit prompts to move<br>• Plan diary<br>• Drink more water<br>• Stand-up for calls<br>• Use coffee app (get free coffee at Patisserie Valarie each day)<br>• Be more mindful<br>• Messages that moving more is good, can transform the body, fidgeting good, limit time seated

		• Centralised kitchen, storage, toilets – go and collect rather than delivery<br>• Encourage others to be active<br>• Be a positive influence for team, show good behaviours and reinforce cultural change<br>• Stand on one foot when making drinks etc.<br>• Drink more water<br>• Understand the links between sitting and health		
**Interpersonal**	• Help support others<br>• Hi-fives<br>• WII games, SNES<br>• Walk to the Stag (pub)<br>• Ping pong<br>• Yoga<br>• Sub-teams – pairs am/pm walks<br>• Paired exercises<br>• Competitions – press-ups etc.<br>• Company dog to walk<br>• Dance competition<br>• All go to gym and bench	• Lunchtime bike or walking group or other activities group<br>• Go out once a week as a group to encourage each other<br>• Work together to help change collective behaviours<br>• Hold team meetings stood up<br>• Team competition or incentive – who's sat least this week (use FitBit / pedometers to measure)?<br>• Challenge the norm of sitting down each meeting<br>• Don't get each other a drink – all get up and make your own	• Encourage others to move about<br>• Activity based team day<br>• Every hour, whole team stands up for 5+ minutes and stretches legs / back / arms<br>• Informal office sports / games<br>• Step competitions – pedometer, daily, weekly, monthly<br>• Walking / running group<br>• Community team group e.g. litter pick<br>• Charity walk<br>• Standing team meetings<br>• 1 day per week, all working at standing desks<br>• Engage / interact with different people in different areas	• Introduce "energisers" to meetings<br>• Build activity into team meetings<br>• Be more diverse with settings for team meetings<br>• Team challenges / competitions on iPhone etc. aim for a certain amount of steps – use colour charts to monitor – red bad, get paler the more active you are

		• Remind one another you've been sat a long time<br>• Support each other – it's ok not to be sat at desk all the time<br>• Booking in standing or walking meetings<br>• Take short breaks – colleagues cover front desk and serve<br>• Outdoor 1:1 meetings	• Teams to learn how to dance	
**Organisational**	• Policy for moving, water policy / wee policy<br>• Impose rules<br>• Change attitude to sitting<br>• Stand up or "get gunged"<br>• Notification to move / stand<br>• Reward for sitting less<br>• Pedometer / FitBit (or individual?)<br>• Organised activities<br>• Bring back PT day<br>• Work upstairs with phones, downstairs LOL<br>• Regular game<br>• Utilise own technology to force<br>• Electrocute chairs if sat for >2 hours<br>• Pay gym memberships<br>• Gaming rehab "operation sit less"	• Celebrate changes / achievements, Tell us your sit less stories<br>• Hot-desking (working in teams) – need support from IT<br>• Change the culture that you are only working at your "station"<br>• Build activity into team meetings<br>• Encourage people to lunch together<br>• Encourage spontaneity from staff<br>• No lunch at desk<br>• Car-free day<br>• Email-free day<br>• No meetings longer than 1 hour<br>• Shorter stand-up meetings	• Raise awareness in terms of health benefits including stress relief<br>• More exercise / health / wellbeing messaging with practical encouragement e.g. 1 day a week no lifts<br>• Financial incentive / flexi to get up for a period throughout the day<br>• Traffic light system for how well we are doing – reward system<br>• Over the tanoy – "Hi di hi!" and stand up<br>• Standing meetings<br>• Walking meetings<br>• Flash up message on Jabber etc.<br>• Encouragement from management	• Extra holidays for logging active hours or other rewards system<br>• Run challenges for points / prizes<br>• Make rewards site easier to access

		• IT system prompt if on PC for over 2 hours (or specified time)<br>• Incentivise stair use, de-incentivise lift use<br>• Incentives – encouragement to walk to other side of building for a snack, reward for walking/steps<br>• Encourage walking / steps competitions<br>• Walk, talk, email – re-order people's responses<br>• Changes in policies and guidelines<br>• Walking and thinking<br>• Give permission so feel able to leave desk<br>• Encourage persistence with initiatives such as walking 1:1s<br>• Programme of standing activities e.g. yoga et<br>• Mobile phones so you don't have to stay at your desks<br>• Invest in mobile technology	• Include motivation around improving health and wellbeing in 1:1s<br>• Guidance and employee support of how to move more e.g. ideas of good stretches to do at your desk<br>• Policy, guidelines, information, suggestions, permission to do<br>• Continue to promote active travel<br>• Make activity breaks as "acceptable" as smoke breaks used to be – less rigid with clear desks policies etc.<br>• Freedom to move around<br>• Maintain the pool cycles better and promote them more – pool cars under 3 or 4 miles, check why not cycled?<br>• Get someone else in to show us how to do it properly e.g. Google type<br>• Change Draconian clear desks policy<br>• Enforced activity break<br>• Exercise classes e.g. Zumba, step class, yoga	
**Environmental**	• Kneel stool<br>• Weighted mice<br>• Wireless / longer headset cable<br>• Office dumb-bells	• Rooftop track (in new building)<br>• High tables to have stand-up meetings	• Under-desk cycling pedals<br>• Standing desks in meeting rooms – poser tables<br>• More standing desks – 1 per bank of 6 desks	• Stability balls for seats<br>• Standing hot-desk, option of standing desk

	• Electric rising desks / varidesk<br>• Standing desks and stool<br>• Standing desks with stool<br>• Supply treadmill desks<br>• Uncomfortable chairs – make you stand-up<br>• Wind-up coffee machine (bike)<br>• Exercise bike<br>• Cross-trainer<br>• Shower room<br>• Waterslide, standing hot tub<br>• Bigger, got to run further<br>• Move closer to the park	• Signage – calories burnt on the stairs, lift sign directing to stairs<br>• More interactive spaces<br>• Outside desk space for own equipment / meetings<br>• More stand-up areas, less chairs<br>• Chairs stacked as standard in meeting rooms or remove chairs – less likely to sit down<br>• Different positioned chairs<br>• Psychology of supermarkets – removing bins, redesign office regularly (teams etc.)<br>• Clear office / desk – get up to get what you need<br>• Storage space for workout gear<br>• External venues for meetings<br>• VR / AR / MR technology – interact with IT<br>• Moving location with arrival of volunteers<br>• Screens on the wall, not desk height<br>• Take wheels off chairs<br>• Gym at work and showers nearby<br>• Activity areas – arm bike, weight machines, pedals, vibrating plates	• Phone change e.g. walk around when talking on the phone<br>• Alternative seating e.g. exercise balls, sit-stand chairs (stools that you can lean on), one uncomfortable chair in each work area<br>• Landscape private outdoor space<br>• Walking route round the office or on the roof<br>• Sporting room e.g. table tennis, snooker, pool, air hockey, Wii, virtual reality<br>• Gym and facilities<br>• Informal exercise area downstairs<br>• Relaxation zone / bean bags	

		• Reduce numbers of stairs<br>• Treadmills around the building<br>• Treadmill desks<br>• Desks that can raise, Higher level desks with no seats, or desks that raise and lower<br>• Hot-desking with standing desks<br>• Standing desks with high chairs		
