# Supplementary material for: Using Co-Production to Develop “Sit Less at Work” Interventions in a Range of Organisations
Source: Int J Environ Res Public Health. 2021 Jul 22;18(15):7751. doi: 10.3390/ijerph18157751 (PMC8345370; doi:10.3390/ijerph18157751)
Supplement: Supplementary file 1 [file ijerph-18-07751-s001.zip › Devel Paper_Table S4.pdf]

Table S4a. Small business 12-week “Sit Less at Work” intervention summary*

Actions to be initiated at the start and to continue throughout the 12-weeks:

- *Throughout: use of social media to promote sit less initiative – TF and MD to oversee, but will delegate some responsibilities to other staff (Inter)*
- *During the team meetings: Included the sit less initiative and develop a policy (working document) – TF to action (Org)*

Week	Action 1	Action 2	Action 3	Action 4	Action 5	Action 6
1	Email from Managing Director (MD) to encourage and support staff – MD to send email (Org**)	Take regular breaks using computer prompt – KE to develop pop-up and provides it to all who want it; all to action (Intra & Org**)	Exercise ball instead of chair – 2-3 x weekly – NE to do rota for use of the ball for duration of 12 weeks; all to action as per rota (Env**)	Do computer games competition which involves movement – KE to ensure all have access to the gaming systems / app, to encourage participation and to ensure all scored appropriately (Inter**)		
2	Email from MD to encourage and support staff	Take regular breaks using computer prompt	Exercise ball instead of chair – 2-3 x weekly	Walk to shops for lunch / snacks – 2-3 x weekly – NE to ask who wants to be involved and then to write a rota for duration of 12 weeks; all to action as per rota (Intra & Org**)	Exercises with office dumbbells – 2-3 x weekly – FH to lead the dumbbell sessions (Inter**)	

**3**	Email from MD to encourage and support staff	Take regular breaks using computer prompt	Exercise ball instead of chair – 2-3 x weekly	Do computer games competition which involves movement– prize awarded at end of week 3	Use wireless headsets to allow walking whilst on the phone – MD to purchase and distribute to staff; all to action<br>(Env**)	
**4**	Email from MD to encourage and support staff	Take regular breaks using computer prompt	Exercise ball instead of chair – 2-3 x weekly	Walk to shops for lunch / snacks – 2-3 x weekly	Exercises with office dumbbells – 2-3 x weekly	Use wireless headsets to allow walking whilst on the phone
**5**	Email from MD to encourage and support staff	Take regular breaks using computer prompt	Exercise ball instead of chair – 2-3 x weekly	Do ping pong competition – 2-3 x weekly – KE to ensure all scored appropriately<br>(Inter)	Use wireless headsets to allow walking whilst on the phone	
**6**	Email from MD to encourage and support staff	Take regular breaks using computer prompt	Exercise ball instead of chair – 2-3 x weekly	Walk to shops for lunch / snacks – 2-3 x weekly	Exercises with office dumbbells – 2-3 x weekly –	Use wireless headsets to allow walking whilst on the phone
**7**	Email from MD to encourage and support staff	Take regular breaks using computer	Exercise ball instead of chair – 2-3 x weekly	Do ping pong competition – 2-3 x weekly – prize awarded at end of week 7	Use wireless headsets to allow walking whilst on the phone	

**8**	Email from MD to encourage and support staff	Take regular breaks using computer prompt	Exercise ball instead of chair – 2-3 x weekly	Walk to shops for lunch / snacks – 2-3 x weekly	Exercises with office dumbbells – 2-3 x weekly	Use wireless headsets to allow walking whilst on the phone
**9**	Email from MD to encourage and support staff	Take regular breaks using computer prompt	Exercise ball instead of chair – 2-3 x weekly	Do press-up competition – 2-3 x weekly – KE to ensure all scored appropriately (Inter)	Use wireless headsets to allow walking whilst on the phone	
**10**	Email from MD to encourage and support staff	Take regular breaks using computer prompt	Exercise ball instead of chair – 2-3 x weekly	Walk to shops for lunch / snack – 2-3 x weekly	Exercises with office dumbbells – 2-3 x weekly	Use wireless headsets to allow walking whilst on the phone
**11**	Email from MD to encourage and support staff –	Take regular breaks using computer prompt	Exercise ball instead of chair – 2-3 x weekly	Do press-up competition – 2-3 x weekly – prize awarded at end of week 11	Use wireless headsets to allow walking whilst on the phone	
**12**	Email from MD to encourage and support staff	Take regular breaks using computer prompt	Exercise ball instead of chair – 2-3 x weekly	Walk to shops for lunch / snacks – 2-3 x weekly	Exercises with office dumbbells – 2-3 x weekly	Use wireless headsets to allow walking whilst on the phone

***** *Individuals responsible for the implementation of each action were highlighted (where the action first appears) on the intervention summary (MD to denote the Managing Director or two random letters to denote other members of staff).*

*** The ecological level that each new action relates to is denoted in each table using the following abbreviations: "Intra" (intrapersonal-level), "Inter" (interpersonal-level), "Org" (organisational-level), and "Env" (environmental-level).*

Table S4b. Small business detailed action plan

Action	Content	Timing	Management involvement	Barriers	How to overcome barriers	Who to action
**Emails from management as a way of encouraging and supporting staff (Org)**	Initial email from MD highlighting the start of the 12 weeks and what staff can expect.<br>Emails throughout the 12 weeks to keep encouraging staff to participate in various initiatives.	Day 1, week 1	Management to take charge of this	Time	Block out time in diary to undertake this task	MD
**Take regular breaks (Intra & Org)**	Set-up a computer prompt which pops-up every hour to remind you to move away from your desk for at least 2 minutes. This can be either a standing or moving break.<br>Pop-up will be a message in a small square box with the project logo on	Day 1, week 1	Approval required from MD via an email sent to staff to give staff permission to participate	Workload pressures may mean that unable to take a break and pop-ups may become annoying / cause frustration	Have the ability to disable pop-ups temporarily	KE to develop pop-up, asks who would like it installed and ensure all who request it are able to access it
**Exercise ball instead of chair (Env)**	Exercise ball – one which rotates around the office, so interested staff can take it in turns to use<br>Each to be rota'd to use it – aim for all interested to use 2-3 x weekly for 2-3 hours at a time<br>Already have an exercise ball in the office	Day 1, week 1	Approval required from MD via an email sent to staff to give staff permission to participate<br>MD/TF to assess health and safety prior to starting	Where to store	Can be kept upstairs when not in use	NE to do a rota for use of the ball (to do whilst also doing lunch pick-up rota)
**Do various activities which involve movement**	1. Play Wii/PS4/SNES games that involve movement	Day 2, Week 1	Approval required from MD via email sent to staff to give staff	Need to ensure no impact on service delivery	Those less keen on taking part in the activities	KE ensure all have access to the gaming

**using some form of competition with a prize (e.g. a bottle of wine) at the end of each month (Inter)**	KE has a spare PS4 system which is already on site and there is an app which can be downloaded for free (phone then acts as the control). Games can involve standing, moving or dancing<br>2. Play ping pong – best of 3 shots (place ping pong table over meeting table)<br>3. Paired exercises e.g. press-ups, boxing – focus on individual competition i.e. how you improve rather than comparing to others	2-3 x weekly in pairs	permission to participate<br>MD/TF to assess health and safety prior to starting	May not include everyone – some may be put off by the idea of competition	could still have a standing break, and do the scoring instead so not being excluded	systems and app<br>MD to purchase prizes<br>KE to encourage participation and ensure all scored appropriately
**Walk to shops for lunch / snacks (Intra & Org)**	People doing the lunch pick-up<br>Take it in turns – using a rota	Day 1, week 2<br><br>2-3 x weekly	Approval required from MD via an email sent to staff to give staff permission to participate	Weather<br>The time to walk to and from the shops would currently be included in the lunch break time so no time to then eat lunch.	Purchase a large umbrella and keep by the front door.<br>For those staff rota'd to do the lunch pick-up – the time required will not be included in lunch break i.e. they can take that time back from the organisation.	NE to ask who wants to be involved and then to write a rota

**Use of social media to encourage staff to participate in the activities but also to advertise the good initiatives (Inter)**	Videos of dancing competitions / exercises – put on Twitter, their website LinkedIn and Facebook<br>Change the staff signatures via signature manager with links to what they're doing<br>Use of "Sit Less at Work" branding	Day 1, Week 2	No specific approval required, just encouragement from management to participate in activities so have something to talk about on social media	Time	Block out time in diaries to undertake this task	TF and MD to oversee, but will delegate some responsibilities to other staff
**Add the "Sit Less at Work" initiative to the team meeting agenda**<br>**Develop a policy (Org)**	Team meetings can be used to discuss how the package is going<br>Use the package of changes document as a working document for the policy which can be added to and developed over time to support sustainability if initiative	First team meeting in 12-week period	Approval for the policy will be required – all staff need to be in favour	No barrier, item is on agenda and staff in agreeance	n/a	TF<br>On agenda for Team Meeting on 11th June
**Wireless headsets to allow walking whilst on the phone**<br>**(Env)**	Wireless headsets (to be purchased) for all who would like them with the aim of being able to stand or walk whilst on the phone	Day 1, week 3	Approval required from MD	Forget to use them	Include a reminder message to use with the computer prompt to remind to break from sitting	MD to purchase and distribute to staff
**Exercises with office dumbbells (Inter)**	Office dumbbells (to be purchased) and exercises to be undertaken	Day 1, week 3<br>2-3 x weekly	Approval required from MD via an email sent to staff to give staff permission to participate	Need to ensure no impact on service delivery<br>May not include everyone – some may be put off by exercise	Those less keen on taking part could still have a standing break, and help count reps so not being excluded	FH to lead the dumbbell sessions

			MD/TF to assess health and safety prior to starting			

Note: the levels of influence each action related to is denoted as follows: "Intra" is intrapersonal, "Inter" is interpersonal, "Org" is organisational, and "Env" is environmental.
