# Supplementary material for: Using Co-Production to Develop “Sit Less at Work” Interventions in a Range of Organisations
Source: Int J Environ Res Public Health. 2021 Jul 22;18(15):7751. doi: 10.3390/ijerph18157751 (PMC8345370; doi:10.3390/ijerph18157751)
Supplement: Supplementary file 1 [file ijerph-18-07751-s001.zip › Devel Paper_Table S7.pdf]

Table S7a. Large corporation 12-week "Sit Less at Work" intervention summary

Week	Action 1	Action 2	Action 3
**1**	Weekly email from Area Manager with sit less suggestions<br>(Intra, Inter & Org*)		
**2**	Weekly email from Area Manager with sit less suggestions	Weekly sit less challenge<br>(Inter & Org*)	
**3**	Weekly email from Area Manager with sit less suggestions	Weekly sit less challenge	
**4**	Weekly email from Area Manager with sit less suggestions	Weekly sit less challenge	Incorporate activity into monthly team meetings<br>(Inter & Org*)
**5**	Weekly email from Area Manager with sit less suggestions	Weekly sit less challenge	
**6**	Weekly email from Area Manager with sit less suggestions	Weekly sit less challenge	
**7**	Weekly email from Area Manager with sit less suggestions	Weekly sit less challenge	
**8**	Weekly email from Area Manager with sit less suggestions	Weekly sit less challenge	Incorporate activity into monthly team meetings
**9**	Weekly email from Area Manager with sit less suggestions	Weekly sit less challenge	
**10**	Weekly email from Area Manager with sit less suggestions	Weekly sit less challenge	
**11**	Weekly email from Area Manager with sit less suggestions	Weekly sit less challenge	
**12**	Weekly email from Area Manager with sit less suggestions	Weekly sit less challenge	Incorporate activity into monthly team meetings

** The ecological level that each new action relates to is denoted in each table using the following abbreviations: "Intra" (intrapersonal-level), "Inter" (interpersonal-level), "Org" (organisational-level), and "Env" (environmental-level).*

Table S7b. Large corporation detailed action plan

Action	Content	Timing	Management involvement	Barriers	How to overcome barriers	Who to action*
**Weekly email from Area Manager with sit less suggestions (Intra, Inter & Org)**	Area Manager to send an email informing team of challenge and importance of sitting less at work. Suggestions on how to sit less to be included in the email: taking the stairs not the lift; if have a FitBit – make sure you do at least 250 steps/hour and make use of prompts (vibration) it makes to do this; drink more water; stand/walk for calls; make use of the coffee app and go get your free daily coffee from Valerie Patisserie – especially on a Friday (when everyone is expected to be in the office) everyone could go together – social element.	Day 1, week 1 and then a weekly email thereafter	Area Manager to send email (template to be written by Researcher)	How to get everyone involved	Use team Whatsapp group to encourage<br><br>Use weekly call on a Monday to prompt/remind	Researcher to write email templates for each week<br><br>TA/EG to check Area Manager happy to send email and encourage participation in weekly meetings
**Weekly sit less challenge (Inter & Org)**	Use the white board next to Area Manager's desk to track everyone's steps (use FitBits or iPhones for step counts) – make it competition with yourself rather than each other – prize for biggest improver rather than who's got most steps.	Day 1, week 2	Approval required from Area Manager	How to get everyone involved	Use team Whatsapp group to encourage<br><br>Use weekly call on a Monday to prompt/remind	TA/EG to get Area Manager approval and set-up & maintain white board progress chart<br><br>Need to decide on prize ?extra holiday days

**Incorporate activity into monthly team meetings (Inter & Org)**	The use of energisers, movement, "treasure hunts" and/or challenges to add activity into meetings.<br>Every other meeting is in an external location – when in an external location, there could be a "treasure hunt" challenge included in the day where team members have to go out and find and take a photo of certain objects e.g. if somewhere with outside space, could take a picture of an insect – the most obscure the better.<br>For external and internal meetings – could incorporate movement into the meeting e.g. coffee break – go and get free coffee from Patisserie Valerie, all team stand/walk on spot for 5 mins every hour etc, who can get the most steps in the lunch break?	Three times during the 12 weeks (at team meeting which occurs once a month)	Area Manager would need to support this and put time in the agenda for activities	Busy agendas, sometimes have guests attending	Stream-line meetings, plan activity in, build it in to break times e.g. lunch / coffee breaks	TA/EG to approach Area Manager to see if happy to include activity into the team meetings<br><br>Area Manager to include into agendas

***** *Individuals responsible for the implementation of each action were highlighted where appropriate (denoted by two random letters).*

Note: the levels of influence each action related to is denoted as follows: "Intra" is intrapersonal, "Inter" is interpersonal, "Org" is organisational, and "Env" is environmental.
